# Origin of the Morgue

**Published:** 1881-02

**Authors:** 


### ORIGIN OF THE MORGUE.

The word morgue had originally a far different meaning from that which it now bears. In old dictionaries it is defined as a surly, haughty look, being derived from the word morguer, to bully, to dare. It is also used (the verb) to express the idea of inspecting or viewing intently and closely any person or object. Madame de Sévigné, in a letter to Madame de Grignan, uses it thus, when she writes: "Guitaut writes me from Saint-Ange three leagues from Fontainebleau, where he has gone to morguer the court." The morgue was originally the second guichet or wicket at the Grand-Châtelet. When newly-arrested prisoners were marched in there, the turnkeys used to halt them in front of this wicket in order that the jailers might morguer them at their ease; that is to say, look them over attentively, and engrave their features in the memory. It was there afterward that the corpses picked up on the public street, or dragged from the Seine, were deposited for recognition. Later, in 1804, there was constructed upon the Quay du Marché-Neuf, at the northeast angle of the Pont Saint-Michel, a square building specially set apart for the exhibition of the unknown bodies. The opening of new boulevards has singularly modified that quarter, and the morgue is to-day removed to the extremity of the Cité, upon that little island a long time since united to the main land, and which was in former days called "La Motte aux Papelards" (The Hillock of the Hypocrites). The exhibition-room consists of two divisions, separated by a glass partition running from the floor to the ceiling, and from one side of the room to the other, through which partition the visitor sees lying on the twelve dalles, or stone forms, the dead bodies, sometimes partially clothed, again entirely naked save a little strip of linen about the waist. One of the most pathetic sights we ever witnessed there was the corpse of a little child not more than three years of age, seated in a "high-chair" just as she was drawn from the river, her baby rattle clutched still in her tiny fingers, her little white cap on her head, and wearing the customary short blouse of the children of the ouvriers. Poor little baby! she had tumbled in the Seine while at play, perhaps while her mother was busily at work in some lavoir near by.

ERRATUM.—In the January number, page 16, twenty-third line from the top, for "stitch" read "stick."

